# Transient Noise Reduction Using a Deep Recurrent Neural Network: Effects on Subjective Speech Intelligibility and Listening Comfort

**DOI:** 10.1177/23312165211041475

**Published:** 2021-10-04

**Authors:** Mahmoud Keshavarzi, Tobias Reichenbach, Brian C. J. Moore

**Affiliations:** 1 Department of Bioengineering and Centre for Neurotechnology, 4615 Imperial College London, London, UK; 2 Centre for Neuroscience in Education, Department of Psychology, 2152 University of Cambridge, Cambridge, UK; 3 Cambridge Hearing Group, Department of Psychology, 2152 University of Cambridge, Cambridge, UK; 4 Department Artificial Intelligence in Biomedical Engineering, Friedrich-Alexander-University Erlangen- Nuremberg, Erlangen, Germany

**Keywords:** hearing aids, transient sounds, speech intelligibility, listening comfort, deep recurrent neural network, machine learning

## Abstract

A deep recurrent neural network (RNN) for reducing transient sounds was developed and its effects on subjective speech intelligibility and listening comfort were investigated. The RNN was trained using sentences spoken with different accents and corrupted by transient sounds, using the clean speech as the target. It was tested using sentences spoken by unseen talkers and corrupted by unseen transient sounds. A paired-comparison procedure was used to compare all possible combinations of three conditions for subjective speech intelligibility and listening comfort for two relative levels of the transients. The conditions were: no processing (NP); processing using the RNN; and processing using a multi-channel transient reduction method (MCTR). Ten participants with normal hearing and ten with mild-to-moderate hearing loss participated. For the latter, frequency-dependent linear amplification was applied to all stimuli to compensate for individual audibility losses. For the normal-hearing participants, processing using the RNN was significantly preferred over that for NP for subjective intelligibility and comfort, processing using the RNN was significantly preferred over that for MCTR for subjective intelligibility, and processing using the MCTR was significantly preferred over that for NP for comfort for the higher transient level only. For the hearing-impaired participants, processing using the RNN was significantly preferred over that for NP for both subjective intelligibility and comfort, processing using the RNN was significantly preferred over that for MCTR for comfort, and processing using the MCTR was significantly preferred over that for NP for comfort.

Hearing aids and cochlear implants employ amplitude compression, also called automatic gain control (AGC), to compress the large range of sound levels encountered in everyday life into the limited dynamic range of impaired human hearing (Dillon, 1996; Keshavarzi, Baer, et al., 2018; Moore, 2008). AGC systems in hearing aids usually filter the incoming signal into several frequency channels and apply AGC to each channel signal independently. The AGC in each frequency channel is characterized by an attack time and a recovery time (ANSI, 2014). When the input sound level abruptly increases, the gain decreases, but this takes time to occur. The time taken for the output to get within 3 dB of its steady value is called the attack time. When the sound level abruptly decreases, the gain increases, but again this takes time to occur. The time taken for the output to increase to within 4 dB of its steady value is called the recovery time or release time. The attack time is often shorter than the release time, so as to avoid discomfort when the sound level increases abruptly. However, despite the use of AGC, users of hearing aids still complain about discomfort and poor speech intelligibility caused by transient sounds such as a door slamming, cutlery clattering, and keys jingling.

Transient sounds are usually characterized by a very fast increase in amplitude (sometimes with a rise time less than 1 ms), a rapid decline (over tens of ms), and a duration of less than a few hundred ms (Dyballa et al., 2015). Although some AGC systems have utilized a separate fast-acting side branch to control the levels of transient sounds (Boyle et al., 2009; Moore et al., 1991; Moore & Glasberg, 1988; Stone et al., 1999), such systems are not fast enough to provide protection from transient sounds with a very fast onset (Dingemanse et al., 2018). Also, if the release time is long, the gain is reduced for some time after the transient has occurred, thereby reducing the audibility and potentially the intelligibility of speech. Finally, if both the attack and release time are made very short, so as to reduce the gain for intense transient sounds, this can lead to reduced overall sound quality (Tan & Moore, 2004). Several transient noise reduction (TNR) algorithms have been developed to mitigate these problems (Digiovanni et al., 2011; Dingemanse et al., 2018; Dyballa et al., 2015, 2016; Hirszhorn et al., 2012; Keshavarzi, Baer, et al., 2018; Korhonen et al., 2013).

Digiovanni et al. (2011) studied the effects of two different TNR algorithms on speech intelligibility and subjective ratings of sound comfort, sound quality, and speech understanding for hearing-impaired (HI) participants. The stimuli were sentences presented in quiet, in multi-talker babble, with two different types of transient (“door slams” and “chair clangs”) added, and using a combination of each type of transient and babble. Each condition was tested with either TNR activated or deactivated, separately for each TNR algorithm. There was an improvement in speech intelligibility with the TNR activated for both algorithms when speech was presented in babble, in the presence of chair clangs (but not door slams), and when babble and chair clangs were combined. However, none of the subjective preference ratings differed significantly for TNR activated and TNR deactivated.

Korhonen et al. (2013) used a paired-comparison task to compare the sound quality and annoyance of impulsive everyday sounds, such as a knife on a plate, a pen tap, and a car door, with a TNR algorithm on versus off. Experienced hearing-aid users clearly preferred the TNR on condition, because the quality of the sounds was less annoying and more natural. Speech intelligibility was not adversely affected by the TNR.

Dyballa et al. (2016) evaluated the effects of a multi-channel TNR algorithm on speech intelligibility and subjective sound quality for cochlear implant users. They found an improvement in reception thresholds for speech in both cafeteria and office noise and higher comfort and clarity ratings for speech in cafeteria noise with the TNR on.

Keshavarzi, Baer, et al. (2018) investigated the effects of a multi-channel TNR algorithm on listening comfort/annoyance for normal-hearing (NH) and HI participants, using three amounts of transient reduction (weak, medium, and strong). For both participant groups, sounds processed using the TNR algorithm were preferred over the unprocessed sounds. Further, the medium and strong settings decreased the annoyance produced by the transient sounds while preserving their audibility.

Overall, while these studies have shown promising results, most of them have not shown that the TNR algorithms improved both speech intelligibility and listening comfort in the presence of transient sounds. Also, when improvements have been found, they have usually been modest. There is clearly room for further improvements.

The systems reviewed above were all based on detecting when a transient had occurred and reduced the gain during the time that the transient was estimated to be present; none were based on the use of neural networks. Over the past few years, artificial neural networks have been widely used in many applications, including hearing and speech processing, and have led to significant advances in these fields. In particular, the use of one of the most successful variants of deep recurrent neural networks, called “long short-term memory” (LSTM, Hochreiter and Schmidhuber, 1997), has been found to be effective in reducing background noise, including wind noise and babble, thus improving the intelligibility and quality of speech in noisy environments for hearing-aid users with mild-to-moderate hearing loss (Keshavarzi et al., 2019; Keshavarzi, Goehring, et al., 2018) and for cochlear implant users (Goehring et al., 2019).

This paper presents a study of transient-sound reduction using a deep (multi-layer) LSTM recurrent neural network (RNN). For brevity, this is hereafter referred to as the RNN. The RNN was first trained to predict the ideal ratio mask (IRM, a soft-gain function based on the ideal Wiener filter in the time frequency [TF] domain) (Delfarah & Wang, 2017; Srinivasan et al., 2006), using recordings of speech corrupted by transient sounds. The clean speech (without transients) was utilized to estimate the IRM. Despite the use of clean speech to estimate the IRM, the goal was not to remove the transient sounds completely, since such sounds convey important information about environmental events. It was assumed that since the trained RNN would not operate perfectly, it would reduce the intensity of the transient sounds without making them inaudible.

Once trained, the RNN was used to process the transient-corrupted speech so as to attenuate TF segments with a low speech-to-transient ratio (STR) while preserving segments with high STR. The effects of the RNN processing on subjective speech intelligibility and listening comfort were assessed for speech in the presence of transient sounds. A multi-channel transient reduction (MCTR) method not based on a neural network (Keshavarzi, Baer, et al., 2018) was used as a comparison condition. The MCTR method has been shown to significantly increase listening comfort for intense transient sounds superimposed on speech for both NH and HI participants (Keshavarzi, Baer, et al., 2018).

## Method

### Participants

Twenty native English-speaking participants took part in the study. None of them had taken part in any of our previous studies. An Amplivox audiometer (International Electrotechnical Commission (IEC) 60645-1, Type 4) was used to measure audiometric thresholds for frequencies from 0.125 to 8 kHz. Ten participants had normal hearing (4 female, average age = 24 years, standard deviation = 3 years), with audiometric thresholds less than 20 dB HL at all audiometric frequencies, and ten participants had mild-to-moderate sensorineural hearing loss. Only the better ear (according to the average audiometric threshold across 0.5 to 4 kHz) of each participant was tested. The gender, age, and audiometric thresholds for the tested ears of the HI participants are given in Table 1. Testing took about two hours for each participant, and participants were paid for taking part and reimbursed for travel costs. The study was approved by the Imperial College Research Ethics Committee.

**Table 1.** Age, Gender, and Audiometric Thresholds (dB HL) of the Hearing-Impaired (HI) Participants.

		Frequency (kHz)							
Gender	Age (years)	0.125	0.5	1	2	3	4	6	8
Female	55	25	25	30	50	55	55	40	50
Female	22	5	15	25	40	55	55	60	50
Female	60	10	25	25	35	10	15	30	50
Male	60	35	30	30	40	55	55	50	45
Female	57	15	20	35	50	55	65	65	70
Female	73	25	25	10	20	35	45	65	65
Male	75	0	20	35	50	60	55	60	65
Female	66	5	10	35	45	50	35	35	45
Male	56	30	30	10	15	30	30	45	70
Female	63	20	15	45	25	15	15	25	15

### Speech Materials and Transients

The speech materials used in the study were taken from the CSTR VCTK (Center for Speech Technology Voice Cloning Toolkit, available at: https://datashare.ed.ac.uk/handle/10283/3443), a British-English multi-speaker corpus created at the University of Edinburgh. We chose this corpus to ensure that the RNN would generalize to a wide range of unseen talkers with different accents. The sentences in the corpus were sampled using a 48-kHz rate with 16-bit resolution. For this study, the sentences were down-sampled to 16 kHz. This was done to be consistent with our previous studies and with our processing toolboxes. A sampling rate of 16 kHz is sufficient to cover the typical frequency range of hearing aids (Moore et al., 2001). Sixteen hundred sentences from 80 talkers (40 female and 40 male) were used for training the RNN and 300 sentences from six other talkers (3 female and 3 male) were used for evaluating the performance of the RNN using objective estimators of speech intelligibility. Twelve sentences were used for subjective evaluations of speech intelligibility and comfort (from two female and two male talkers, three sentences for each talker), randomly taken from the 300 sentences used for the objective measures. The sentences had a mean duration of 1.98 s, with a standard deviation of 0.59 s.

Twenty-four different transients were used. Fifteen transients (described in Table 2) were used to train the RNN and nine (described in Table 3) for evaluating the RNN using objective measures and for the experimental evaluation. Note that some of the transients, such as a bag of bottles breaking, contained multiple peaks. On each trial, one transient was added to one sentence at a randomly determined position within the sentence. The STR was calculated as the ratio of the root-mean-square (RMS) level of the clean speech relative to the RMS level of the transient measured in a 5-ms rectangular window centered around the peak amplitude of that transient. STRs of −5, −10, and −15 dB were used for training the RNN. STRs of −5, −10, −15, and −20 dB were used for objective evaluation of the RNN. STRs of −10 and −15 dB were used for the subjective evaluations. These STRs were chosen so that the transients would be at least somewhat unpleasant in the condition of no processing (NP).

**Table 2.** Transient Sounds Used for Training the Recurrent Neural Network (RNN).

A concrete block hit with a metal hammer
A metal can filled with metal bolts, shaken once
A plastic ball-point pen being clicked
A metal spoon being swirled in a porcelain cup
A glass vase hit with the finger
Automatic gun fire in the distance
Knocking on a door
Opening of a door
A church bell
A window breaking
A wine bottle breaking
Hammer and chisel on brick
Hammering of a brick wall
Hammering an iron stake into masonry
Laying a table in preparation for a meal

**Table 3.** Transient Sounds Used for Testing the Recurrent Neural Network (RNN) and for Evaluating the Subjective Effects.

Two water glasses tapped together
A glass jar filled with glass marbles, shaken once
A set of keys dropped on a wooden table
Two metal rails hit together
A knife being flicked with the fingernail
Milk bottles breaking
Bag of bottles breaking
Desk bell ringing
A closing door

### RNN Algorithm

**Figure 1.**
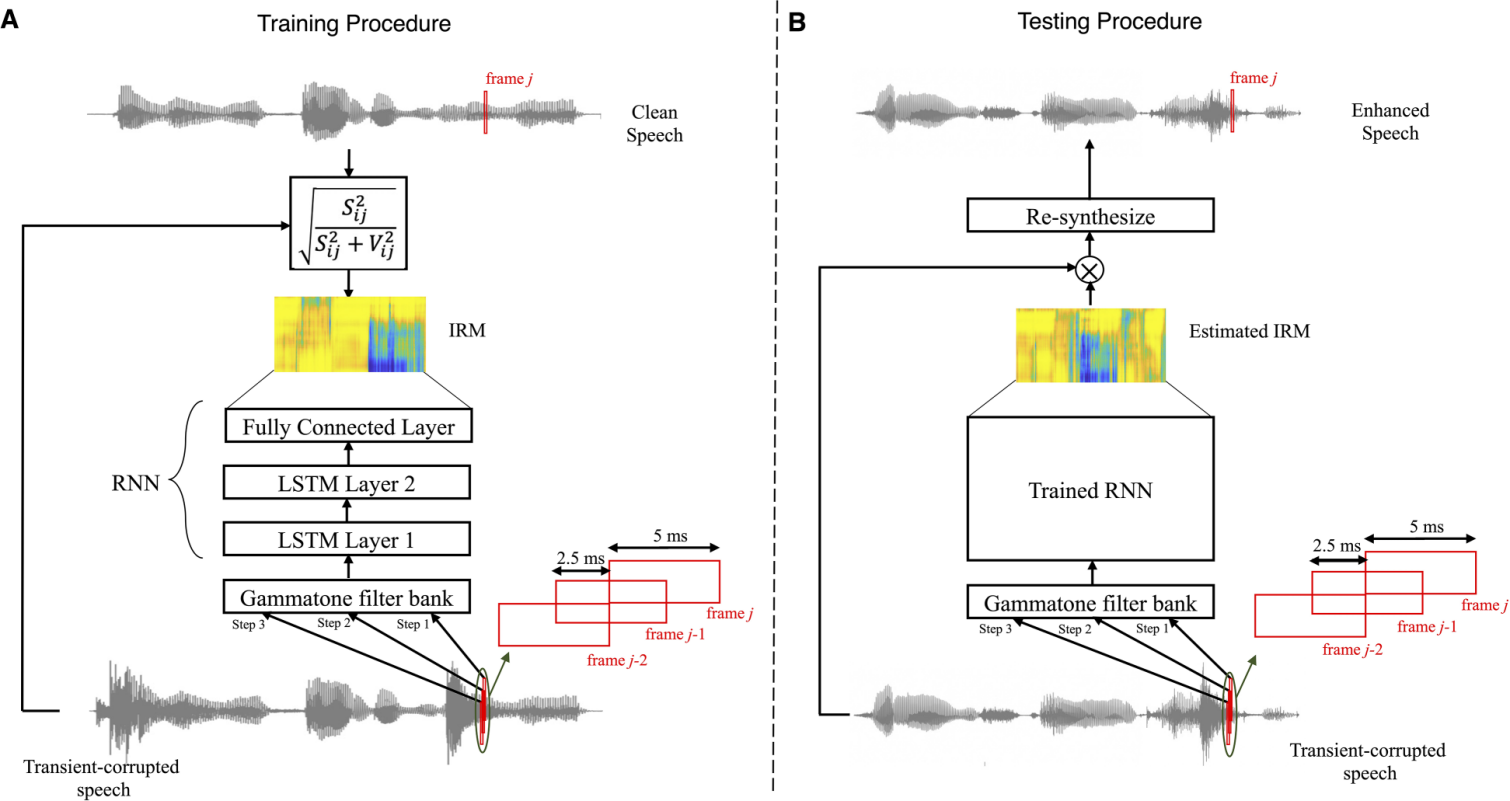
Schematic diagram of the RNN algorithm used in this study. Panels A and B show the training and testing procedures, respectively.

Figure 1 shows schematic diagrams of the training of the RNN (part A) and the application of the trained RNN (part B). The input signal (bottom), namely the speech corrupted by a transient sound, was modeled as:

(1) x(t)=s(t)+v(t)

where *t* is time, *x* is the corrupted speech, *s* is the clean speech, and *v* is the transient sound. The RNN consisted of an input layer, two LSTM layers with 128 and 64 units, respectively, and a fully connected output layer with 64 units. The RNN processed three-time-step inputs, where each step corresponded to features extracted from a single time frame of speech; steps 1, 2, and 3 corresponded to successive time frames *j*−2, *j*−1, and *j*, respectively.

The acoustic features used to train the RNN were the energy in each time frame at the output of a 64-channel gammatone filter bank (Patterson et al., 1995) with filter center frequencies equally spaced on the ERB_N_-number scale (Glasberg & Moore, 1990) and ranging from 50 to 8,000 Hz. The gammatone features were calculated using a fast Fourier transform with 5-ms Hanning-windowed time frames with a 50% overlap. Acoustic features were feed into the RNN as the inputs and the IRM was predicted as the output. The IRM for the *i*th frequency band and *j*th time frame, $IRM_{ij}$ , was defined as (Delfarah & Wang, 2017):

(2) $$IRM_{ij}=\sqrt{\frac{S_{ij}^{2}}{S_{ij}^{2}+V_{ij}^{2}}}$$

where $S_{ij}$ and $V_{ij}$ are the magnitudes of *s*(*t*) and *v*(*t*) in the *i*th frequency channel of time frame *j*, respectively.

The clean speech was used to obtain $S_{ij}$ and to calculate the IRM during training. The objective of the training was for the IRM estimated by the RNN to be as close as possible to the true IRM. The machine learning frameworks “Keras” (Chollet, 2015) and “Tensorflow” (Abadi et al., 2016) were used to build, train and test the RNN. The “Adam” optimizer (Kingma & Ba, 2014) with learning rate = 0.001, *β*_1_ = 0.9, *β*_2_ = 0.999, *ε* = 10^−8^ was used as the optimizer method during training so as to minimize the mean square error. The batch size was 1,500 and 5 training runs (epochs) were used. Although there are some similarities between the RNN used in the present study and the ones employed in our previous studies, such as the number of layers, the type of input features (Gammatone features), and the target type (IRM), they differ in terms of the number of time steps, the number of units in each layer, batch size, and the number of epochs.

After the RNN had been trained, it was used to estimate the IRM for each TF segment in each time frame. The estimated IRM was used to process the noisy speech in each time frame so as to attenuate each TF unit by an amount depending on the estimated STR for that TF unit; the lower the STR, the greater was the attenuation, according to equation 2. The overlap-add procedure (Allen, 1977) was used to reconstruct the complete signal from the processed overlapping time frames.

### MCTR Algorithm

**Figure 2.**
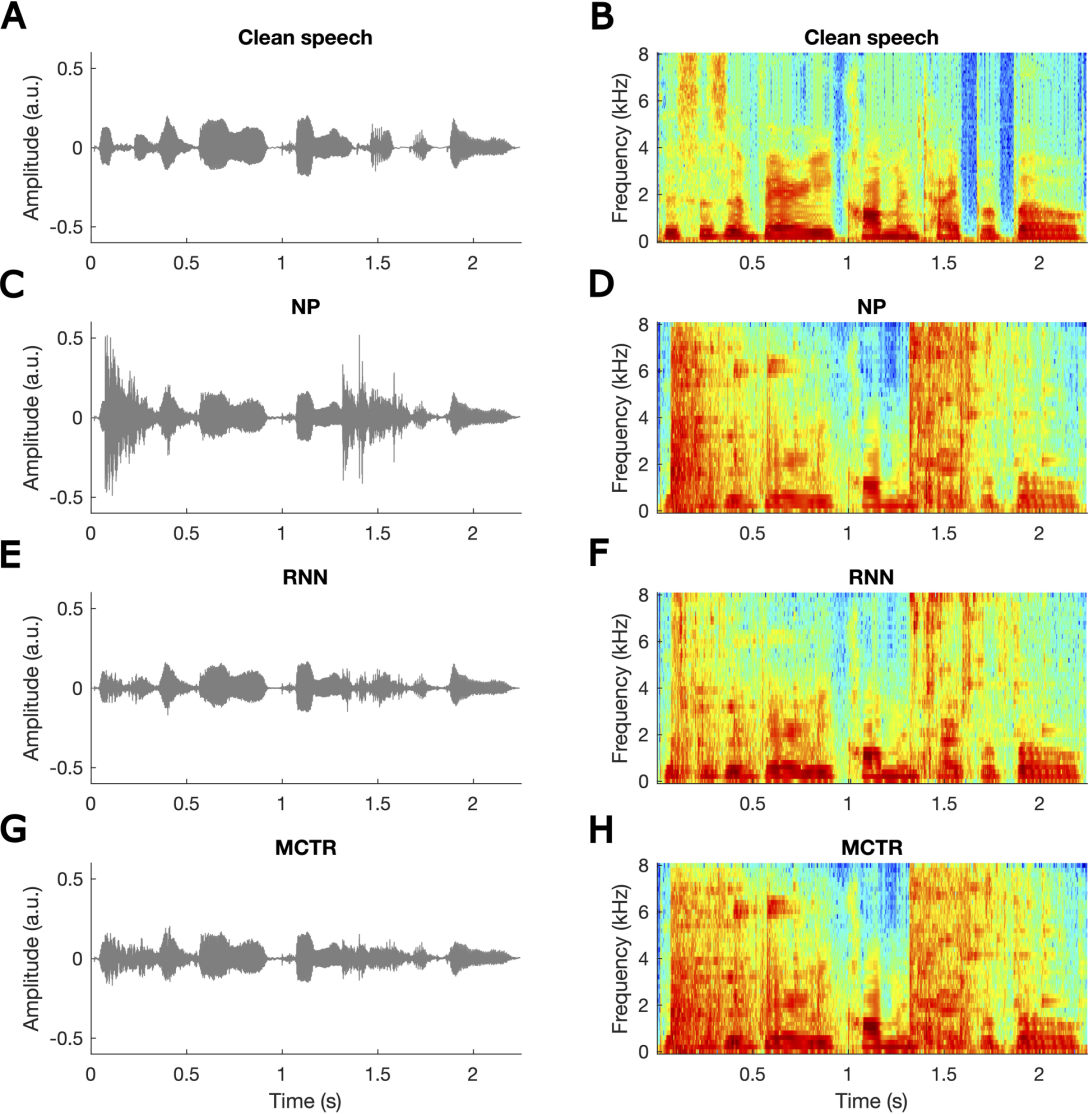
Waveforms (left) and spectrograms (right) of clean speech (panels A and B), speech corrupted by two transients (panels C and D), corrupted speech processed by the RNN (panels E and F), and corrupted speech processed by the multi-channel transient reduction (MCTR) algorithm (panels G and H). Note that two transients are shown purely for illustrative purposes; only one transient per sentence was used during training and testing.

The MCTR algorithm (Keshavarzi, Baer, et al., 2018) was used as a comparison condition. The MCTR was not based on a neural network. It used seven steps to reduce transient sounds: (1) the input signal was resampled to 22.05 kHz; (2) the resampled signal was segmented into 1-ms (22 samples) time frames with a 12-sample overlap, using a Tukey window; (3) a frequency-domain representation of each time frame was calculated by applying a 32-point FFT to the signal in each time frame, resulting in 16 frequency bins; (4) the frequency bins were grouped into 5 frequency channels. The number of bins in frequency channels 1 to 5 was 1, 1, 2, 3, and 9, respectively; (5) Transients were detected by comparing the short-term magnitude in frequency channel *i* and time frame *j*, $M_{ij}$ , to a running estimate of the RMS magnitude in that frequency channel and that time frame, $RMS_{ij}$ . A transient was deemed to be present in frequency channel *i* of time frame *j* when:

(3) $$M_{ij}/RMS_{ij}\;>\;\delta_{i}$$

where the values $\delta_{i}$ were 12, 21, 12, 8, and 7 for frequency channels 1 to 5, respectively; (6) the magnitude for the *i*th frequency channel of that time frame was attenuated by an amount, *C_ij_*, whose value in dB was defined by:

(4) $$C(R_{ij})=\left\{\begin{array}{c} \alpha R_{ij}\;\;\;\;\;\;\;\;\;\;\;\;\;\;\;R_{ij}>0\;\;\;\;\;\;\;\;\;\;i=1,\;2,\;\ldots ,\;5\\ 0\;\;\;\;\;\;\;\;\;\;\;\;\;\;\;\;\;\;\;\;\;otherwise\;\;\;\;\;\;\;\;\;\;\;\;\;\;\;\;\;\;\;\;\;\;\;\;\;\;\;\;\; \end{array}\right.$$

where α was 0.467 and $R_{ij}$ was $20\mathrm{lo}g_{10}(M_{ij}/RMS_{ij})$ ; (7) the processed signal was down-sampled to 16 kHz. The value of α corresponds to the “medium” setting used by Keshavarzi, Baer, et al. (2018). Figure 2 shows both time domain waveforms (left) and spectrograms for the clean speech, speech corrupted by two transients (one near the start of the speech and one centered at about 1.4 s after the start), corrupted speech processed using the RNN, and corrupted speech processed by the MCTR algorithm. Note that two transients were used in the figure for illustrative purpose only; only one transient per sentence was used for training and testing of the RNN.

### Procedure

The participant was seated in a soundproof room and wore a Sennheiser HD650 headset connected to the sound card of a Macbook Pro. The HD650 headphones have approximately a diffuse-field response, and levels were specified as equivalent diffuse-field levels based on measurements made with a KEMAR dummy head (Burkhard & Sachs, 1975). The stimuli for the HI participants were given linear frequency-dependent amplification according to the “Cambridge formula” (Moore & Glasberg, 1998) to ensure that the speech was audible over a wide frequency range. This was done separately for each HI participant, based on the audiogram of the test ear, using a 513-tap finite impulse response filter implemented using the fir2 function in MATLAB (the Mathworks). Three conditions were used: NP, RNN, and MCTR. For condition NP, the RMS input level of the speech (excluding the transient sounds and prior to the Cambridge-formula amplification for the HI participants) was 60 dB sound pressure level (SPL). Since the RNN and MCTR attenuated the transient level markedly and the speech level only slightly, the overall level of the processed speech-plus-transient was set to 60 dB SPL. This level was chosen to be consistent with our previous study (Keshavarzi, Baer, et al., 2018); it led to a comfortable level for the speech while preventing the transient from being excessively loud. The three conditions were compared in terms of subjective intelligibility and comfort, using the paired-comparison procedure described by Moore & Sęk, (2013). There were three pair-wise comparisons: RNN versus NP, RNN versus MCTR, and MCTR versus NP. The two sounds to be compared were presented in succession with a 1-s silent interval between them. Both of the two possible orders were used for each pair and the order was randomized across trials.

The main experiment consisted of two separate parts. In the first part, the participant was asked to indicate their preference in terms of subjective speech intelligibility. The instructions to the participant for this part, which appeared on the computer screen, were the same as those used by Keshavarzi et al. (2019): “On each trial you will hear the same sentence twice in succession. Please decide whether the first or second sentence is more intelligible and by how much, by using the mouse to position the slider on the screen.”

In the second part, the participant indicated their preference in terms of listening comfort. The instructions for this part were same as those used by Keshavarzi, Baer, et al. (2018): “On each trial, you will hear the same sentence twice in succession. A transient background sound (e.g., the sound of glasses clinking) has been added to each sentence. The background sound should be clearly audible and it should sound natural, but it should not be too loud or too annoying and it should not interfere with your perception of the sentence. Please decide whether you prefer the sound in the first interval or the sound in the second interval, and by how much, by using the mouse to position the slider on the screen. Your judgment should be based on the balance between the audibility/naturalness of the transient sound and its loudness/annoyance. For example, if the transient sound is barely audible or does not sound natural in the first interval and is clearly audible and natural but not too loud or annoying in the second interval, you should indicate a preference for interval 2. On the other hand, if the sound is clearly audible and natural in both intervals, but is comfortably loud in interval 1 and louder or more annoying in interval 2, you should indicate a preference for interval 1.”

On each trial, each pair of stimuli was presented only once. The participant responded using a mouse to move a slider on the screen along a continuum labeled “1 much better,” “1 moderately better,” “1 slightly better,” “equal,” “2 slightly better,” “2 moderately better,” and “2 much better.” Choices were not limited to the labeled positions; any position along the slider could be chosen. In each part of the study, each of the three pairs of conditions was presented twice in both orders for each of twelve sentences and for two STRs (–10 dB and –15 dB). Therefore, there were 144 trials in each part.

Preference scores for each participant and each pair of conditions were calculated as described by Moore and Sęk (2013). The extreme positions of the slider were arbitrarily assigned values of −3 and +3. Regardless of the order of presentation of two conditions, X and Y, if condition X was preferred, the slider position was assigned a negative number and if condition Y was preferred the slider position was assigned a positive number. The overall score for a particular comparison and a given STR was obtained by averaging the scores obtained for both orders for that comparison at that STR for each participant. Scores were then averaged separately for the NH and HI participants.

## Results

### Objective Evaluation of Speech Intelligibility

**Figure 3.**
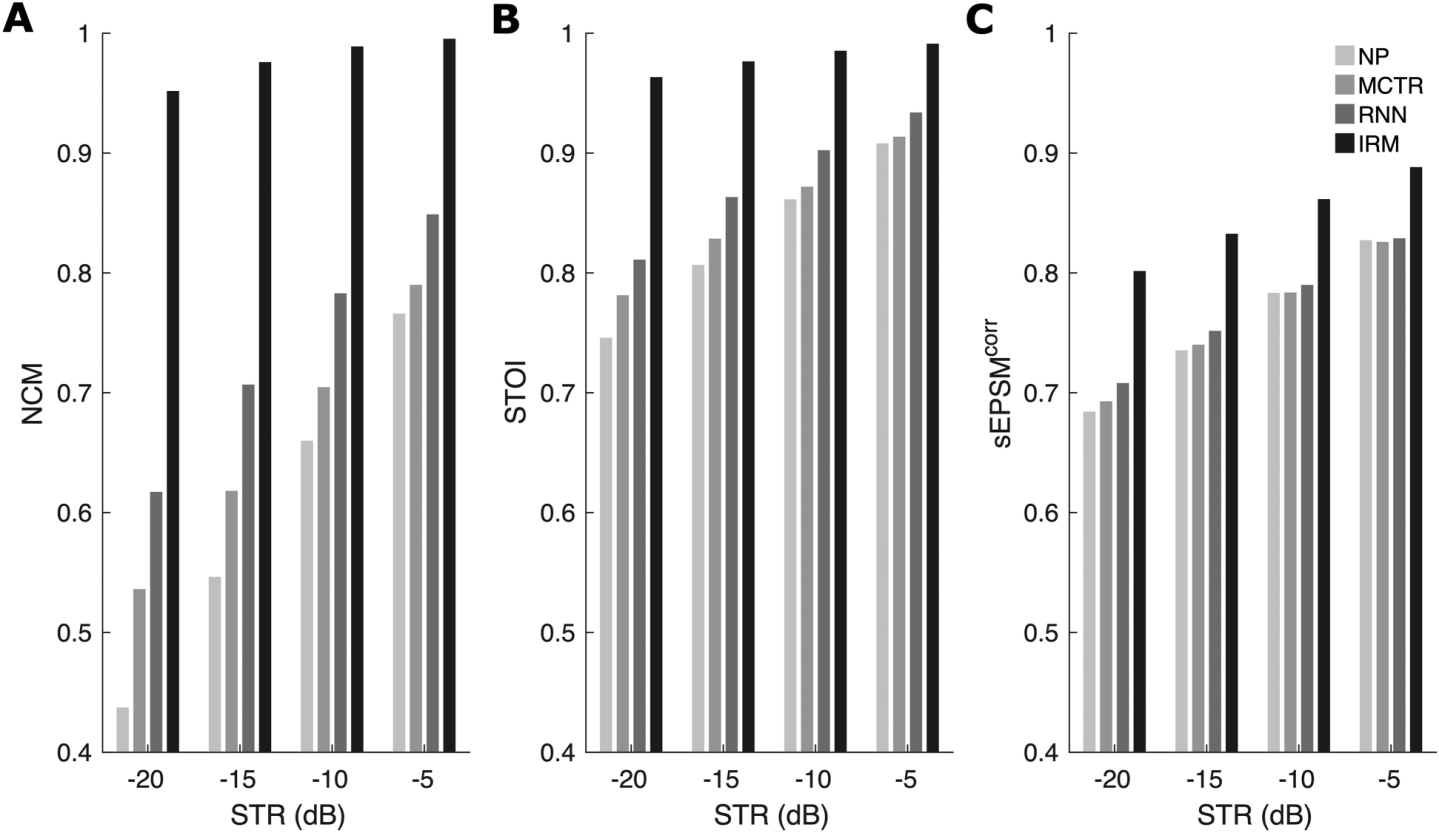
Objective measures of speech intelligibility (NCM, panel A; STOI, panel B; sEPSM^corr^, panel C) for transient-corrupted speech processed using NP, MCTR, RNN, and true IRM at STRs of −5, −10, −15, and −20 dB. Higher numbers indicate greater predicted intelligibility.

As a check that the RNN and MCTR algorithms were performing in a reasonable way and were not markedly distorting the speech, three objective metrics were used to estimate speech intelligibility for the stimuli used in conditions NP, MCTR, and RNN. The metrics were the normalized covariance metric (NCM, Ma et al., 2009), the short-time objective intelligibility (STOI, Taal et al., 2011), and the sEPSM^corr^ (Relano-Iborra et al., 2016). All metrics use the clean speech as a reference and all give values ranging from 0 to 1, where 0 indicates very poor intelligibility and 1 indicates very high intelligibility. All metrics are based on filtering the signal into frequency channels and assessing the similarity of the channel envelopes for the original signal and the corrupted signal. The metrics were calculated for 300 sentences from six talkers. Figure 3 shows the results for the NCM (panel A, left), STOI (panel B, middle), and sEPSM^corr^ (panel C, right) for conditions NP, MCTR, RNN, and also for the ideal case of stimuli processed using the true IRM, for STRs of −5, −10, −15, −20 dB. As expected, all metrics decreased with decreasing STR, although the decrease was small for IRM processing. Importantly, all three metrics gave higher values for condition RNN than for conditions MCTR and NP, especially for the lower STRs, although the effect was small for sEPSM^corr^. All metric values were lower for the RNN than for the true IRM, indicating that the RNN was less than perfect in estimating the IRM, as expected.

MCTR = multi-channel transient reduction; NCM = normalized covariance metric; STOI = short-time objective intelligibility; NP = no processing; RNN = recurrent neural network; STR = speech-to-transient ratio.

### Preferences Scores for Intelligibility

To assess whether the preference scores for a given paired comparison and a given STR were significantly different from zero (indicating a significant preference for one condition relative to another at that STR), the scores for each participant were first averaged across the twelve sentences and two orders of presentation used for the evaluation. Shapiro-Wilk tests showed that the scores were not normally distributed for some pairs of conditions, so Wilcoxon signed-rank tests were used to assess whether the ten resulting scores (one for each participant) differed significantly from zero (using two-tailed tests). This was done separately for each pair of conditions and each STR and separately for the NH and HI participants. No correction for multiple comparisons was applied because we were testing specific hypotheses that both RNN and MCTR processing would be preferred over NP.

**Figure 4.**
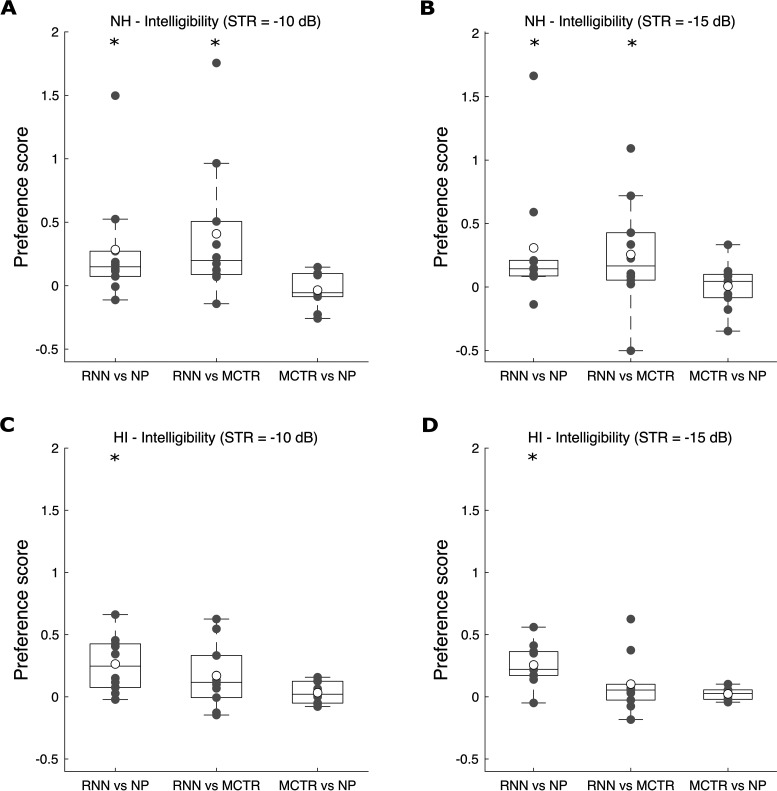
Box and whisker plots of preference scores for speech intelligibility for each pair-wise comparison. Panels A and B show the results for the NH participants and panels C and D show results for the HI participants. Open circles denote the average preference scores across participants and the gray symbols show the individual scores.

Note. *symbols indicate preference scores that are significantly different from zero.

Figure 4 shows box plots of the preference scores for speech intelligibility for each STR (−10 dB left and −15 dB right) and each pair of conditions, for the NH (top) and HI (bottom) participants. For each pair of conditions, if the score fell above 0, this indicated that the first condition in the pair was preferred. For example, for the column labeled RNN vs NP, the mean preference score was above 0, so condition RNN was on average preferred over condition NP. The average preference scores for all pairs of conditions were small (below 1, corresponding to the “slightly better” label on the slider), especially for the HI participants.

NH = normal-hearing; HI = hearing-impaired.

For the NH participants (Figure 4A and B): (1) RNN was significantly preferred over NP for both STRs (*W* = 4, *p* = .014 for STR = –10 dB; *W* = 4, *p* = .014 for STR = –15 dB); (2) RNN was significantly preferred over MCTR for both STRs (*W* = 4, *p* = .014 for STR = –10 dB; *W* = 8, *p* = .049 for STR = –15 dB); (3) There was no significant preference for MCTR vs NP for STR = –10 dB (*W* = 24, *p* = .77) or STR = –15 dB (*W* = 25, *p* = .85).

For the HI participants (Figure 4C and D): (1) RNN was significantly preferred over NP for both STRs (*W* = 1, *p* = .004 for STR = –10 dB; *W* = 1, *p* = .004 for STR = –15 dB); (2) There was no significant preference for RNN vs MCTR for STR = –10 dB (*W* = 11, *p* = .11) or for STR = –15 dB (*W* = 15, *p* = .23); (3) There was no significant preference for MCTR vs NP for STR = –10 dB (*W* = 13, *p* = .26) or STR = –15 dB (*W* = 13, *p* = .16).

### Preferences Scores for Listening Comfort

**Figure 5.**
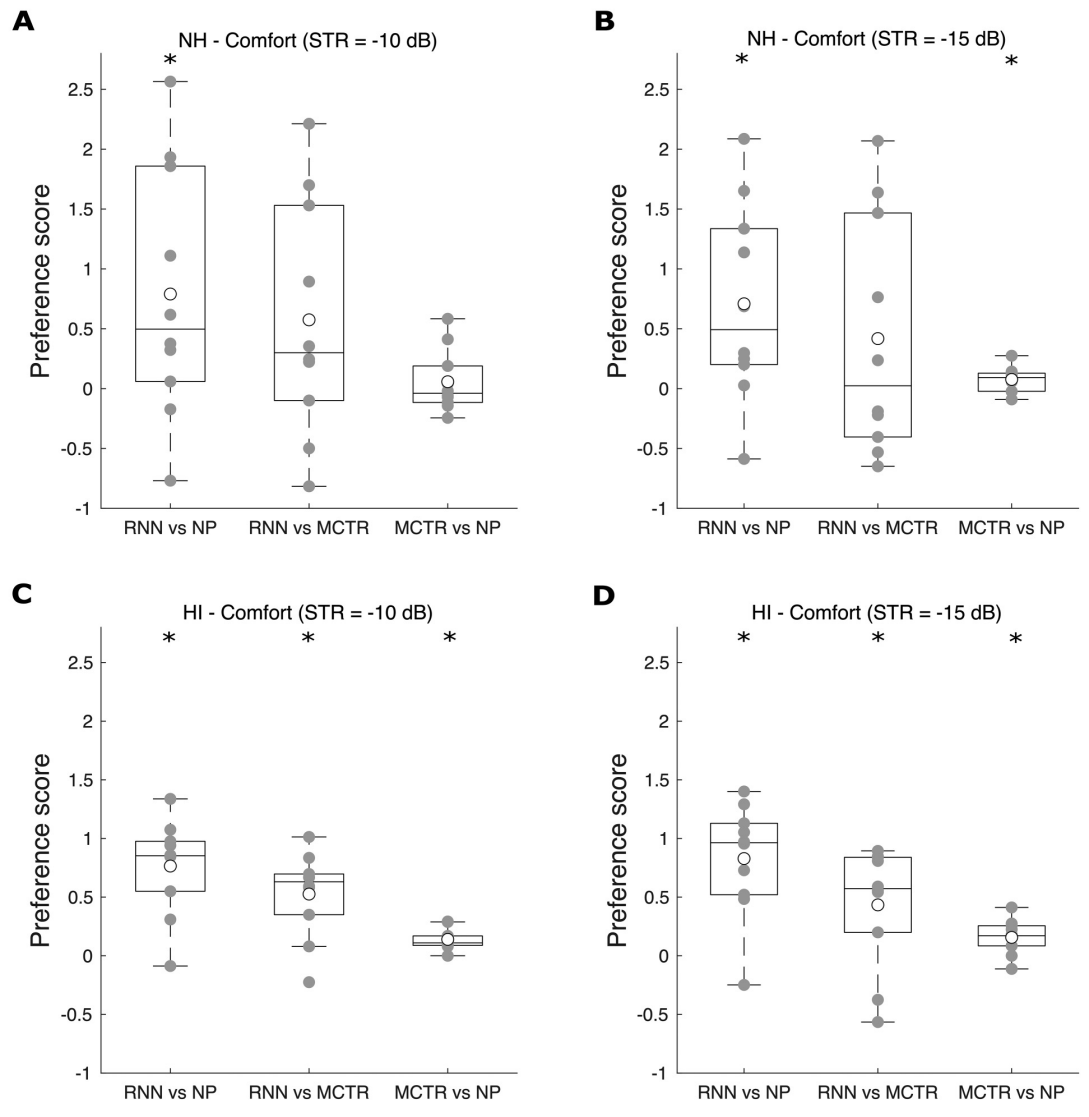
As Figure 4, but for listening comfort.

Figure 5 shows box plots of the preference scores for listening comfort. The average preference scores for all pairs of conditions were again small, although the preferences for the HI participants for listening comfort were generally larger than for intelligibility. For the NH participants (Figure 5A and B): (1) RNN was significantly preferred over NP for both STRs (*W* = 8, *p* = .049 for STR = –10 dB; *W* = 5, *p* = .02 for STR = –15 dB); (2) RNN was not significantly preferred over MCTR for either STR, although there was a trend in that direction (*W* = 12, *p* = .13 for STR = –10 dB; *W* = 18, *p* = .38 for STR = –15 dB); (3) There was no significant preference for MCTR vs NP for STR = –10 dB (*W* = 27, *p* = 1), while there was a small but significant preference for MCTR vs NP for STR = –15 dB (*W* = 8, *p* = .049).

For the HI participants (Figure 5C and D): (1) RNN was significantly preferred over NP for both STRs (*W* = 1, *p* = .004 for STR = –10 dB; *W* = 1, *p* = .004 for STR = –15 dB); (2) RNN was significantly preferred over MCTR for both STRs (*W* = 2, *p* = .006 for STR = –10 dB; *W* = 7, *p* = .037 for STR = –15 dB); (3) MCTR was significantly preferred over NP for both STRs (*W* = 0, *p* = .008 for STR = –10 dB; *W* = 3, *p* = .021 for STR = –15 dB).

### Preference Scores for Individual Transients

**Figure 6.**
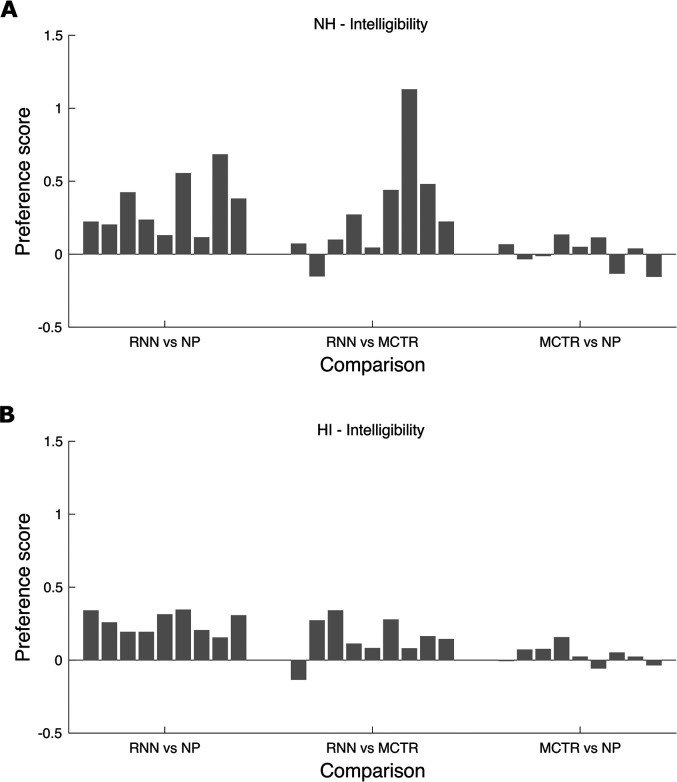
Preference scores for subjective intelligibility for each transient for each pair-wise comparison, averaged across participants within each group and across the two speech-to-transient ratios (STRs). The ordering of the nine transients (from left to right) is the same as in Table 3.

To assess the consistency of preference scores across the nine transients, the scores for each transient were averaged across participants for each pair-wise comparison. To reduce the effects of random variability, the scores were also averaged across the two STRs. The results for subjective intelligibility are shown in Figure 6. For the RNN vs NP comparison, the preference scores were small but positive (indicating a preference for RNN over NP) for all of the transients, for both the NH and HI participants. For the RNN vs MCTR comparison, the preference scores were positive for all of the transients except transient 2 (a glass jar filled with glass marbles, shaken once) for the NH participants and transient 1 (two water glasses tapped together) for the HI participants, indicating a preference for RNN over MCTR for most transients. For the MCTR vs NP comparison, the preferences were very small and varied in sign across transients. In summary, there were consistent preferences for intelligibility across transients for RNN over NP and mostly consistent preferences for RNN over MCTR.

**Figure 7.**
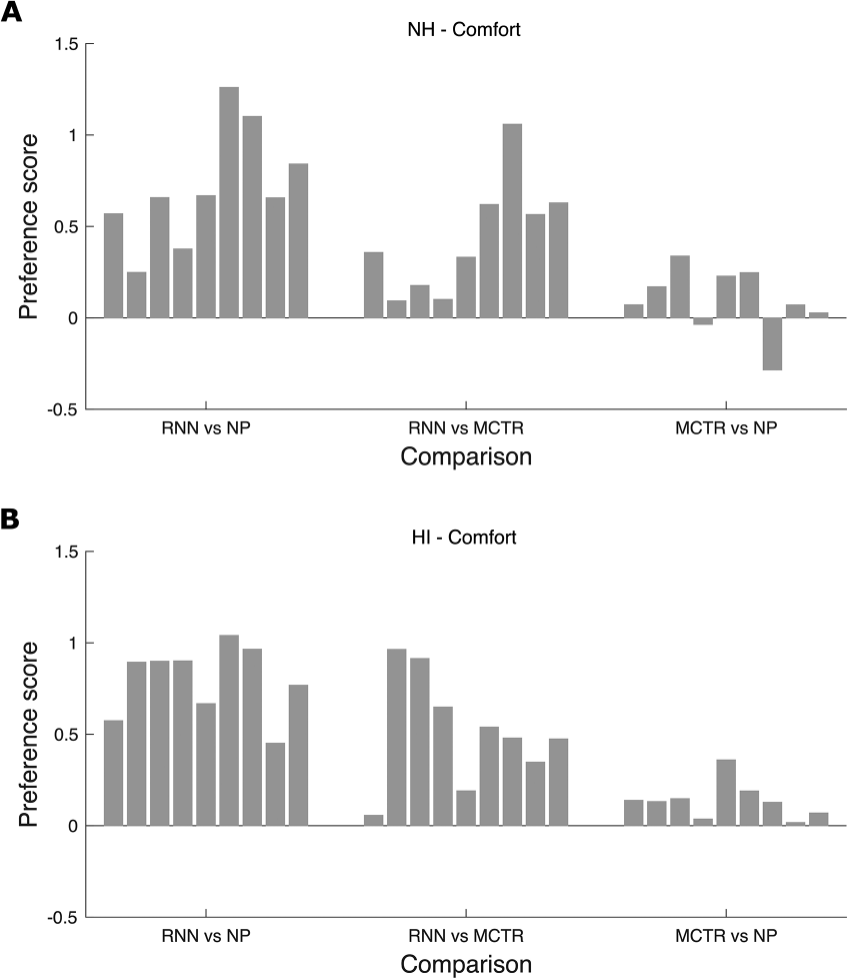
As Figure 6, but for listening comfort.

The results for comfort are shown in Figure 7. For the RNN vs NP comparison, the preference scores were positive (indicating a preference for RNN over NP) for all of the transients, for both the NH and HI participants. For the RNN vs MCTR comparison, the preference scores were again positive (indicating a preference for RNN over MCTR) for all of the transients, although the preference score was close to 0 for transient 1 for the HI participants. For the MCTR vs NP comparison, the preferences were very small but were mostly positive. In summary, there were consistent preferences for comfort across transients for RNN over NP and for RNN over MCTR.

## Discussion

The evaluation using the objective predictors of intelligibility (NCM, STOI, and sEPSM^corr^) showed that the RNN led to higher scores than NP, especially for the lower STRs used. However, the objective scores for the RNN processing were not as high as for processing based on the true IRM. While this indicates that the RNN was not as effective as the IRM in attenuating the transients, it should be borne in mind that, as anticipated, the RNN did not remove the transients completely. In real life, people need to be aware of events going on around them, and it is important that sounds like a door slamming remain audible after attenuation. That was the reason why the instructions in terms of listening comfort included the sentence “Your judgment should be based on the balance between the audibility/naturalness of the transient sound and its loudness/annoyance.” It appears that while the RNN was not actually trained to keep the transient sounds audible, it did this at least to some extent. It would be interesting in a future study to train the RNN not using the clean speech as a reference, but rather using speech corrupted by transients with higher STRs, such that the transients were audible but not uncomfortable.

To assess generalization, the RNN was tested using unseen talkers and unseen transients. All three objective measures and some of the subjective results indicated that at least some generalization did occur. However, it is not known how well the RNN would generalize for speech in the presence of background sounds such as babble, in addition to transients. That is a topic for future studies.

In a previous study evaluating the MCTR algorithm (Keshavarzi, Baer, et al., 2018), three settings of α were used, 0.267, 0.467, and 0.933 corresponding to weak, medium, and strong transient reduction (attenuation). Preferences were evaluated only for listening comfort, i.e., in terms of the balance between the annoyance produced by the transient and the audibility of the transient; the instructions were the same as for the present study. The results for the NH participants indicated that MCTR processing was preferred over NP and that the medium attenuation setting was slightly preferred over the weak attenuation setting and the strong attenuation setting. The results for the HI participants also showed a preference for MCTR processing over NP, but the medium and strong attenuation settings were preferred over the weak attenuation setting. These results justified the use of the medium attenuation setting for both groups in the present study. The results of the present study for listening comfort for MCTR vs NP were similar to those of the earlier study, in showing small but significant preferences for MCTR.

The results of the present study showed that for listening comfort and the HI participants, RNN processing was significantly preferred over NP and over MCTR processing for both STRs (Figure 5). In other words, the RNN processing improved listening comfort more than the MCTR processing. The preference for RNN processing over NP contrasts with the results of Digiovanni et al. (2011) who found that preferences did not differ significantly with their TNR system active versus inactive. Our results are consistent with those of Korhonen et al. (2013) and Dyballa et al. (2016), in showing subjective preferences for the conditions with TNR activated. It is difficult to compare the magnitude of the benefit of the TNR systems across studies because of differences in the transients used and in the STRs used.

For subjective speech intelligibility and for the HI participants, RNN processing was significantly preferred over NP, but the preference for RNN over MCTR was not significant, although there was a trend for RNN to be preferred (Figure 4). It would be desirable in a future study to measure speech intelligibility via listening tests, rather than gathering subjective preferences in terms of intelligibility, although this might require more difficult speech materials, to avoid ceiling effects.

The preference scores were generally small, although for the HI participants and for listening comfort at −15 dB STR, the median score for RNN versus NP reached 1. The small preference scores may reflect the fact that the stimulus levels were chosen to avoid highly aversive loudness. Higher preference scores might have been obtained if higher overall levels or lower STRs had been used. The small obtained scores might also reflect the effects of random variability in judgments, and the reluctance of participants to use the extremes of the rating scale. The preferences for both intelligibility and comfort were consistent across transients for the RNN vs NP comparison and reasonably consistent across transients for the RNN vs MCTR comparison, indicating that the RNN worked well with the different types of unseen transients.

The processing delay produced by the RNN processing was restricted to a value that would be acceptable for users of hearing aids. To achieve this, the RNN processed time frames with a duration of 5 ms and with an overlap of 2.5 ms. With a fast processor, the delay produced by the RNN in estimating the IRM would be negligible, so the overall delay caused by the RNN processing would be about 7.5 ms, which is below the maximum acceptable value for hearing aids for closed fittings (Stone & Moore, 1999; Stone & Moore, 2005) but slightly above the maximum acceptable value for open fittings (Stone et al., 2008). Thus, the RNN processing could be applied in hearing aids and cochlear implants, especially when a closed fitting is used for the former. Also, the RNN processing could be implemented so as to work in parallel with other processing, such as dynamic range compression and noise reduction. This means that the RNN would not necessarily increase the total processing delay of the hearing aids or cochlear implants.

## Limitations of the Study

There were several limitations of our study: (1) Since a standard set of transient sounds does not exist, it is difficult to compare our results with those obtained using other TNR algorithms; (2) Due to covid-related restrictions, we were limited to only one experimental session (lasting about 2 h) during one visit for each participant. This limited the number of trials per transient for each STR, leading to more “noisy” data than would be obtained with more trials; (3) Only ten participants with normal hearing and ten with mild-to-moderate hearing loss were tested. It would be desirable to test more participants to increase statistical power and to establish whether preferences for TNR depend on the degree and pattern of hearing loss; (4) The RNN processing was evaluated only using transients added to speech in quiet. Further work is needed to train and evaluate an RNN for transient reduction when other background sounds are also present; (5) We obtained subjective preferences for speech intelligibility rather than measuring intelligibility using listening tests. It would be desirable in the future to measure speech intelligibility in listening tests for speech corrupted by transient sounds in combination with different types of background sound.

## Summary and Conclusions

An RNN for reducing the loudness and annoyance of transient sounds was trained using sentences spoken with different accents and corrupted by a variety of transient sounds, using the clean speech as the target. The RNN processing was tested using sentences spoken by unseen talkers and corrupted by unseen transient sounds to ensure that the processing generalized appropriately. A paired-comparison procedure was used to compare all possible combinations of three conditions in terms of subjective speech intelligibility and listening comfort for two relative levels of the transients, −10 and −15 dB. These STRs were chosen so that the transients would be at least somewhat unpleasant when NP was applied. The conditions were: NP; processing using the RNN; and processing using a MCTR not based on an RNN.

Ten participants with normal hearing and ten with mild-to-moderate hearing loss participated. For the latter, frequency-dependent linear amplification using the Cambridge formula was applied to all stimuli to compensate for individual audibility losses. For the NH participants, processing using the RNN was significantly preferred over that for NP for subjective intelligibility and comfort, processing using the RNN was significantly preferred over that for MCTR for subjective intelligibility, and processing using the MCTR was significantly preferred over that for NP for comfort for the higher transient level only. For the HI participants, processing using the RNN was significantly preferred over that for NP for subjective intelligibility, processing using the RNN was significantly preferred over that for MCTR and for NP for comfort, and processing using the MCTR was significantly preferred over that for NP for comfort.

Overall, the results indicate that the RNN processing was more effective in improving listening comfort than the MCTR processing evaluated previously.
